# POLYPHARMACY, FRAILTY, AND DISABILITY-FREE SURVIVAL IN COMMUNITY-DWELLING HEALTHY OLDER INDIVIDUALS

**DOI:** 10.1093/geroni/igac059.2122

**Published:** 2022-12-20

**Authors:** A R M Saifuddin Ekram, Robyn Woods, Joanne Ryan, Sara Espinoza, Julia Gilmartin-Thomas, Raj Shah, Mark Nelson, Michael Ernst

**Affiliations:** Monash University, Nunawading, Victoria, Australia; Monash University, Melbourne, Victoria, Australia; Monash University, Melbourne, Victoria, Australia; Sam and Ann Barshop Institute, UT Health San Antonio, San Antonio, Texas, United States; Victoria University, Melbourne, Victoria, Australia; Rush University, Chicago, Illinois, United States; Menzies Research Institute, University of Tasmania, Hobart, Tasmania, Australia; The University of Iowa, Iowa City, Iowa, United States

## Abstract

**BACKGROUND:**

Polypharmacy and frailty are two common geriatric syndromes. We examined the association between polypharmacy and frailty and if, together, they predicted disability-free survival (DFS), defined as time to the first event of dementia, persistent physical disability or death.

**METHODS:**

We included 19,114 participants from the "ASPirin in Reducing Events in the Elderly" (ASPREE) clinical trial. Polypharmacy was defined as regular, concomitant use of five or more prescription medications. Frailty was assessed using a modified Fried phenotype and a deficit accumulation frailty index (FI) of 66 items. The association between polypharmacy and frailty was assessed by multinomial logistic regression. In addition, Cox regression was used to determine the association between polypharmacy-exposed frailty and DFS.

**RESULTS:**

Individuals with polypharmacy (vs. < 5 medications) were 55% more likely to be pre-frail (Relative Risk Ratio or RRR: 1.55; 95%Confidence Interval or CI:1.44, 1.68) and three times more likely to be frail (RRR: 3.34; 95%CI: 2.64, 4.22) according to Fried phenotype. Frail individuals had a two-fold reduction in their survival free of dementia/disability (Hazard ratio or HR: 2.16; 95%CI: 1.56, 2.99), whereas frail individuals with polypharmacy had a four-fold reduction (HR: 4.24; 95%CI: 3.28, 5.47). Effect sizes were more prominent when frailty was assessed using the FI than when assessed by Fried phenotype.

**CONCLUSION:**

Polypharmacy was significantly associated with pre-frailty/frailty. Polypharmacy-exposed pre-frailty/frailty increased the risk of death, dementia or physical disability among older adults. Addressing polypharmacy in older people could ameliorate the impact of frailty on individuals’ functional status, cognition and survival.

